# Health-related quality of life in patients with Barrett’s esophagus

**DOI:** 10.1186/s12955-016-0551-2

**Published:** 2016-11-14

**Authors:** Chi-Yang Chang, Lukas Jyuhn-Hsiarn Lee, Jung-Der Wang, Ching-Tai Lee, Chi-Ming Tai, Tao-Qian Tang, Jaw-Town Lin

**Affiliations:** 1 Department of Internal Medicine, E-Da Hospital, I-Shou University, No.1, Yi-Da Rd., Kaohsiung, 824 Taiwan; 2 School of Medicine and Big Data Research Centre, Fu Jen Catholic University, No.510, Zhongzheng Rd., Xinzhuang Dist., New Taipei City, 24205 Taiwan; 3 National Health Research Institutes, National Institute of Environmental Health Sciences, No.35, Keyan Rd., Zhunan Township, Miaoli County 35053, Taiwan; 4 Department of Environmental and Occupational Medicine, National Taiwan University Hospital, No.7, Chung Shan S. Rd., Taipei, 10002 Taiwan; 5 Department of Neurology, National Taiwan University Hospital, No.7, Chung Shan S. Rd., Taipei, 10002 Taiwan; 6 Institute of Occupational Medicine and Industrial Hygiene, College of Public Health, National Taiwan University, No.7, Chung Shan S. Rd., Taipei, 10002 Taiwan; 7 Department of Public Health, National Cheng Kung University Hospital, College of Medicine, National Cheng Kung University, No. 1, University Rd., Tainan, Taiwan; 8 Department of Internal Medicine, National Cheng Kung University Hospital, No. 1, University Rd., Tainan, Taiwan; 9 Department of Environmental and Occupational Medicine, National Cheng Kung University Hospital, No. 1, University Rd., Tainan, Taiwan; 10 School of Medicine, Fu Jen Catholic University, No.510, Zhongzheng Rd., Xinzhuang Dist., New Taipei City 24205 Taiwan

**Keywords:** Barrett’s esophagus, Quality of life, GERD

## Abstract

**Background:**

Gastroesophageal reflux disease (GERD) has become a major health problem globally, affecting patients’ health-related quality of life (HRQOL). Barrett’s esophagus (BE) is a precancerous lesion associated with GERD. BE patients might not only suffer from HRQOL losses by GERD but also face psychological distress due to the increased risk of developing cancer. However, the majority of patients in Asia have shorter BE segment which is different from the West. This study aimed to determine whether the HRQOL in BE patients were worse than in healthy referents in Taiwan.

**Methods:**

Patients who received referral esophagogastroduodenoscopy for various symptoms were evaluated for the existence of BE. Lesions were judged as endoscopically suspected esophageal metaplasia (ESEM) if they showed morphological resemblances to BE by endoscopy. The diagnosis of BE was confirmed by histology with intestinal metaplasia or gastric metaplasia based on the Montreal definition. The World Health Organization Quality of Life (WHOQOL-BREF) was administered to BE patients before treatment. For each BE patient, we selected 2 age-, sex-, educational background and municipality-matched healthy referents, sampled by simple randomization method from a national survey in Taiwan. Multiple linear regression models were constructed to control the potential confounders.

**Results:**

A total of 84 patients diagnosed with BE were enrolled as BE group and then compared with 168 healthy referents. The BE group had significantly lower WHOQOL-BREF scores than those of healthy referents in the physical domain (*P* < 0.05) but higher scores in the environment domain (*P* < 0.05). In the physical domain, the BE group had significantly lower scores in various facets, including pain, discomfort, sleep and rest and dependence on medications or treatments. There was no significant difference in social and psychological domains between the BE group and healthy referents.

**Conclusions:**

BE patients suffer from poor sleep and rest and high dependence on medications, which significantly reduce their quality of life. Individual facets of each domain warrants a better clinical healthcare to improve quality of life of BE patients.

## Background

Gastroesophageal reflux disease (GERD) has become a major health problem globally [1–4]. Patients with GERD usually suffer from various symptoms, including heart burn, acid regurgitation, epigastralgia, non-cardiac chest pain, chronic cough, asthma and hoarseness. Nighttime acid regurgitation symptoms may interfere with sleep. Therefore, patients with GERD may experience losses on their health-related quality of life (HRQOL) compared with the healthy population [5–7]. Barrett’s esophagus (BE) involves intestinal metaplastic changes of esophageal squamous mucosa, which is regarded as a precancerous lesion of esophageal adenocarcinoma [8]. The development of BE is associated with GERD [9]. The reported prevalence of BE in Western countries varied from 6.3 to 13.6% in patients with GERD [5, 10–12].

Patients with BE often share similar symptoms as patients with GERD [8, 13]. These symptoms could affect their HRQOL [14–19]. Patients who experienced a longer duration of GERD symptoms or higher grade of erosive reflux disease (ERD) had higher risk of developing BE [3, 10, 20–22]. However, 17–40% of BE subjects didn’t report reflux symptoms [3, 23–25]. The difference of QOL between GERD patients and BE patients remains inconsistent. Some studies indicated no significant difference between these two groups [16, 17]. In contrast, Lippmann et al. found that BE patients have better HRQOL than patients with non-erosive reflux disease (NERD) or ERD [15]. This difference is only partially attributable to fewer severe symptoms among BE patients. Under the stress of increased cancer risks, BE subjects might present with a poorer score of QOL in psychological domain. However, psychological distress did not seem to differ significantly between GERD and BE patients [15]. Gerson et al. did not detect a significant difference in time-trade off (TTO) utility values based on heartburn symptoms or annual risk of cancer in patients with non-dysplastic BE [18]. However, TTO utility values are significantly lower for BE subjects with increasing cancer risks such as BE patients with lower-grade dysplasia or high-grade dysplasia.

To have a fair determination of QOL in BE patients, we should compare them with a healthy representative referents and control of potential confounders. However, few previous studies of the QOL of BE patients could fulfill these criteria. Moreover, QOL should cover not only physical and psychological health but also social and environmental status (e.g., home environment, social support, financial resource and transport). In 1991, the World Health Organization initiated a project to develop a generic and standardized QOL instrument simultaneously in many countries, which led to the World Health Organization Quality of Life (WHOQOL) instrument [26]. The WHOQOL has two unique features. First, it encompasses physical, psychological, social and environment domains. Second, it is a cross-cultural instrument developed for use across different patient groups in different countries [27]. The WHOQOL Group further developed a simplified questionnaire, called the WHOQOL-BREF [28]. The WHOQOL-BREF is also a sensitive tool to evaluate HRQOL of patients with different diseases [29–31].

Considering that most Asian patients have shorter BE segment compared to patients in Western countries, this study aimed to determine whether HRQOL of BE patients were worse than healthy referents in the ethnic Chinese population in Taiwan, adjusted for potential confounding factors.

## Methods

### Patients

Patients who received esophagogastroduodenoscopies (EGD) at E-Da Hospital from April 1, 2009 to March 31, 2012 were recruited into this study.. Lesions were judged as endoscopically suspected esophageal metaplasia (ESEM) if they showed morphological resemblances to BE by endoscopy [32]. The circumference and maximum diameter of BE were rated according to the Prague C and M criteria. The length of BE less than 3 cm was defined as short segment BE. A standardized endoscopic biopsy protocol (i.e., a random biopsy from four quadrants, every 2 cm) was performed at sites with ESEM. The diagnosis of BE was confirmed by histology based on the Montreal definition and classification [32]. All patients with newly diagnosis BE were enrolled in this study. Body weight and height were recorded. The presence of diabetes, hypertension, heart disease, cancer and other major diseases, as well as education level, marital status, employment, religion, monthly income and histories of smoking or drinking were recorded. The Institutional Ethics Committee of E-Da Hospital approved this study (EMRP-098-093).

### HRQOL questionnaire

All subjects were asked to complete a validated generic QOL questionnaire (WHOQOL-BREF, Taiwan version) in the outpatient clinic prior to medical treatment. The Taiwan version of the WHOQOL-BREF contains four domains (physical, psychological, social and environment), including the 26 original items of the WHOQOL-BREF, plus two culture-specific questions. One item addressing “respect from others” was categorized into the social domain, and another corresponded to “eating what one likes to eat” and was categorized to the environment domain. The method of application, the scoring procedures and reference time point (during the last 2 weeks) were the same as the original WHOQOL-BREF [28]. In brief, each item was scored from 1 to 5 points, and a higher score was considered a better QOL. Because the numbers of items are different for each domain, the domain scores were calculated by multiplying the average scores of all items in the domain by a factor of 4. Therefore, each domain score would have the same range, from 4 to 20.

### Reference population

A reference group with sex, age (within 3 years), municipality, marriage and education background-matched healthy subjects was randomly sampled from the database of 2001 National Health Interview Survey (NHIS) conducted by the National Health Research Institute and the Bureau of Health Promotion, Department of Health, Taiwan [33]. The 2001 NHIS was intended to provide nationwide estimates on health conditions, health behaviors and distribution of medical resources for the Taiwanese population. The WHOQOL-BREF, Taiwan version, was one of the tools included in this national survey program. In total, 27,160 eligible persons living in 7357 households were selected through multi-stage sampling proportional to household population size in January 2001. This data is very unique in that it is considered representative of the national population in terms of age, sex and urbanization index. In our study, each BE patient was matched with two reference subjects from the national sample.

### Statistical analysis

We conducted a descriptive analysis to compare the demographic characteristics and each domain of WHOQOL between BE patients and reference subjects using *T*-test.. We further used multiple linear regression models to estimate the summary scores of each domain and individual items as dependent variables, while the presence of BE, BE with dysplasia, BE length, age, sex, years of education, employment, monthly income, marital status, smoking and alcohol drinking were included as the independent predictive variables. A forward stepwise strategy was applied to select significant independent variables with *P* < 0.05 as the inclusion criterion. All data were collected and analyzed using SAS version 9.2.

## Results

A total of 84 BE patients were diagnosed by EGD and histological confirmation during the study period. Among these BE patients, 56 (66.7%) reported GERD associated symptoms, 51 (60.7%) were diagnosed as erosive esophagitis, 68 (81.0%) had short segment BE, and only 7 (8.3%) had low-grade dysplasia.

Table 1 shows the demographic and clinical characteristics of 84 patients with BE and 168 matched healthy referents. The mean age of BE patients was 54.1 years and 82.1% of them were male. Compared to healthy subjects, BE patients had higher prevalence of smoking and drinking and higher body mass index (BMI).

**Table 1:** Demographic characteristics and domain scores of patients with Barrett’s esophagus, and age-, sex-, municipality-, marriage- and education-matched healthy referents

Characteristics	Barrett’s esophagus (*n* = 84)	Healthy referents (*n* = 168)	*P* Value
Sex (% male)	82.14	82.14	1.0
Age (mean ± SD)	54.11 ± 14.29	53.17 ± 14.36	0.98
% Married	80.95	77.38	0.52
% Education (>12 years)	22.62	22.02	0.92
% Employment	83.33	74.07	0.11
% Smoking*	36.90	22.96	0.03
% Drinking*	57.14	22.96	<0.001
BMI (kg/m^2^) (mean ± SD) *	25.42 ± 3.42	22.44 ± 2.91	<0.001
Q1 overall QOL*	3.15 ± 0.80	3.36 ± 0.63	0.028
Q2 overall health*	2.92 ± 0.84	3.57 ± 0.69	<0.001
Physical*	12.42 ± 1.57	15.14 ± 2.42	<0.001
Psychological	13.44 ± 1.86	13.67 ± 2.27	0.43
Social	14.45 ± 2.11	14.22 ± 2.41	0.45
Environment*	14.53 ± 2.04	13.71 ± 2.31	0.006

* *P* < 0.05

### Multiple linear regression analysis of HRQOL scores in BE patients and healthy subjects

To improve statistical efficiency, the educational status was classified as higher educational background (>12 years) and lower educational background (≤12 years). Low socio-economic status was defined as subjects with monthly income less than 667 US dollars. Results of multiple regression analysis for different domain scores of WHOQOL-BREF showed that BE patients had lower scores in the physical domains and higher scores in the environment domain (Table 1). However, QOL scores in the psychological and social domain were similar between the two groups. BE patients had lower scores of overall QOL and health than the healthy referents. Marriage was the major factor associated with increased HRQOL scores (Table 2). Higher educational background and high age were associated with increased scores in the environment domain.

**Table 2:** Significant regression coefficients and standard error (in parentheses) based on multiple linear regression analysis of HRQOL and determinants in patients with Barrett’s esophagus, and age-, sex-, municipality-, marriage- and education-matched healthy referents

	Physical	Psychological	Social	Environment
Constant	14.58** (0.33)	12.81** (0.32)	13.40** (0.35)	11.12** (0.64)
BE (yes/no)	−1.78** (0.22)	-	-	1.01** (0.29)
Marriage (yes/no)	0.65* (0.33)	0.94* (0.36)	1.11* (0.39)	-
Age (year)	-	-	-	0.042** (0.012)
Education (>12 years/≤12 years)	-	-	-	0.81* (0.35)
Sex (female/male)	-	-	-	-
Employment (yes/no)	-	-	-	-
Drinking (yes/no)	-	-	-	-
Smoking (yes/no)	-	-	-	-
BMI (kg/m^2^)	-	-	-	-

* *P* < 0.05; ** *P* < 0.005

### Multiple linear regression analysis of HRQOL scores in facets of each domain

Table 3 summarizes results of multiple linear regression analysis of HRQOL scores in facets of each domain, after adjusting for potential confounding factors. In the physical domain, BE patients had significantly lower scores in pain and discomfort, sleep and rest and dependence on medication or treatments. In the environment domain, BE patients also had higher scores in various facets, including financial resources, physical safety and security, home environment, health and social care, physical environment, opportunities for acquiring new information and skills, transport and eating.

**Table 3:** Regression coefficients and standard error (in parentheses) based on multiple linear regression analysis of each facet of HRQOL in patients with Barrett’s esophagus, and age-, sex-, municipality-, marriage- and education-matched healthy referents

Domains	Facets	BE (yes/no)	Age (year)	Sex (female/male)	Marriage (yes/no)	Drinking (yes/no)	Smoking (yes/no)	BMI (kg/m^2^)	Education (>12/≤12 years)
Physical	Pain and discomfort	−1.81** (0.12)	-	-	-	0.32* (0.14)	−0.31* (0.14)	-	-
	Energy and fatigue	-	0.0093* (0.0046)	−0.39* (0.14)	-	-	-	-	-
	Sleep and rest	−0.72** (0.13)	-	-	-	-	-	-	-
	Mobility	-	-	-	0.38* (0.13)	-	-	-	0.27* (0.13)
	Activities of daily living	-	-	−0.28* (0.12)	0.25* (0.12)	-	-	-	-
	Dependence on medication or treatments	−2.31** (0.13)	-	-	-	-	-	-	-
	Working capacity	-	-	-	-	-	-	-	0.27* (0.11)
Psychological	Thinking, learning, memory & concentration	-	0.0097* (0.0048)	−0.45* (0.15)	-	-	-	-	-
	Self-esteem	-	-	−0.28* (0.12)	-	-	-	-	-
	Body image & appearance	-	-	−0.30* (0.15)	-	−1.32** (0.20)	−1.35** (0.12)	-	-
	Negative feelings	−0.89** (0.12)	−0.0098* (0.0049)	-	-	-	-	-	-
	Positive feelings	0.27* (0.12)	-	-	-	-	-	-	-
	Spirituality/religion/personal beliefs)	0.32* (0.12)	-	-	0.45** (0.15)	-	-	-	-
Social	Social support	-	-	−0.23* (0.11)	0.25 (0.11)	-	-	−0.030* (0.014)	-
	Personal relationships	0.21* (0.10)	-	-	-	-	-	-	-
	Sexual activity	-	−0.0096* (0.0041)	-	0.63** (0.13)	-	-	−0.032* (0.014)	-
	Being respected & accepted	-	-	−0.25* (0.12)	-	-	-	-	-
Environment	Financial resources	0.29* (0.13)	0.014* (0.0055)	-	-	-	-	-	0.46** (0.16)
	Physical safety and security	0.29* (0.11)	0.011* (0.0042)	-	-	-	-	-	-
	Home environment	0.24* (0.10)	0.013** (0.0037)	-	-	-	-	-	-
	Health and social care: availability and quality	0.35** (0.092)	-	-	-	-	-	-	-
	Physical environment	0.27* (0.12)	0.018** (0.0048)	−0.29* (0.15)	-	-	-	-	-
	Opportunities for acquiring new information and skills	0.33* (0.13)	-	-	0.30* (0.15)	-	-	−0.037* (0.019)	0.45** (0.14)
	Participation in & opportunities for recreation or leisure	-	0.015* (0.0057)	-	-	-	-	-	0.41* (0.17)
	Transport	0.28** (0.092)	0.0088* (0.0038)	-	-	-	-	-	0.27* (0.11)
	Eating	0.38** (0.10)	-	-	-	-	−0.24* (0.11)	-	-

* *P* < 0.05; ** *P* < 0.005

We found that marriage was associated with higher HRQOL scores in facets of mobility, activities of daily living, spirituality/religion/personal beliefs, social support and sexual activity, opportunities for acquiring new information and skills, and eating. A higher education level was associated with higher HRQOL scores in facets of mobility, working capacity, financial resources, opportunities for acquiring new information and skills, participation in and opportunities for the recreation or leisure, and transport. In the environment domain, higher age was associated with higher HRQOL scores in facets of financial resources, physical safety and security, home environment, physical environment, participation in & opportunities for recreation or leisure and transport.

## Discussion

Although many studies [14–18] reported significantly lower scores of QOL among BE patients, none of them controlled for potential confounding factors comprehensively. Most studies focused on the difference of QOL between patients with GERD and BE [15–18] but lacked a comparison with normal population. Eloubeidi et al. [17] conducted a prospective study to compare the HRQOL between BE and GERD patients but didn’t find significant difference between these two groups. A generic QOL questionnaire, SF-36, was applied to test the difference between the GERD, BE subjects and age-matched normal referents from the U.S. Patients with GERD or BE had lower QOL scores in all subscales of SF-36 than the general US population. Kuliq et al. [16] compared the HRQOL among patients with NERD, ERD and BE using a prospective cohort study design, but didn’t find significant difference among these three groups. However, all of them had lower QOL scores of SF-36 than the age and gender-matched normal referents in Germany. However, neither Eloubeidi nor Kuliq’s studies controlled potential confounders. Multiple linear regression model has been applied by various QOL related researches to control confounding factors [34–36]. As age, sex, marriage, drinking, smoking, BMI and education could partially explain variations of scores of items and domains of WHOQOL (Tables 2 and 3), these factors might potentially confound the findings of previous studies. Furthermore, the referents sampled from the US and German studies did not represent the nationwide population. Therefore, the difference between BE and normal population was still un-settled.

To our knowledge, our study is the first one which included nation-wide healthy referents and also adjusted for potential confounding factors, including age, sex, education, municipality and marriage. In particular, the healthy referents were randomly sampled from a nationwide population in Taiwan. The WHOQOL-BREF was one of tools in this survey. After controlling for potential confounding factors, we have demonstrated that BE patients suffered from poor QOL in physical domain and its associated various facets, but higher QOL scores in environment domain and its associated facets (Tables 2 and 3). The psychological and social domains were not affected by BE. Case-control study is an important method to find the difference between patients with specific disease and healthy group. However, QOL could be influenced by different culture, region and country. Therefore, the inclusion of a nation-wide healthy subjects as our control group is the strength of this study. Some of QOL studies for variable diseases from Asia use nation-wide healthy referents, such as irritable bowel syndrome by Jamali et al., epilepsy by Liou et al. [34], lung cancer by Lee et al. [37, 38], obesity by Chang et al. [30].

Previous studies on HRQOL of BE patients reported the negative impacts on physical and mental scales based on the SF-36 [16, 17], which is a generic instrument. We administered the WHOQOL-BREF in this study, which is also a generic QOL questionnaire with coverage extended to items of social, and environment domains. In the physical domain, BE subjects had poorer QOL in facets of pain and discomfort, sleep and rest and dependence on medication or treatments. These affected facets make sense empirically and can raise attention in clinical practice when treating BE patients. Our finding that BE patients had higher QOL scores in environment domain were consistent with a previous HRQOL study [37]. In Taiwan, patients with some major chronic diseases can access medical treatment easily owing to its high coverage of National Health Insurance. For BE patients in Taiwan, medical treatment, such as proton pump inhibitor and regular EGD surveillance, have been covered by the national insurance. In addition, BE associated information can be easily acquired by patients from health system or media. Therefore, higher QOL scores were reported in many facets of the environment domain, such as financial resources, physical safety and security, home environment, health and social care, physical environment, transport, opportunities for acquiring new information and skills, etc.

The WHOQOL-BREF encompasses physical, psychological, social and environment domains and various facets associated with each domain. It is a generic questionnaire covering broad fields of QOL. Our study revealed the patients with BE in Taiwan had poor HRQOL in physical domain but better in environment domain. WHOQOL-BREF is a sensitive tool to evaluate HRQOL for patients with different diseases, such as diabetes mellitus [39], tuberculosis [40], lung cancer [37], inflammatory bowel disease [41], irritable bowel syndrome [42, 43], morbid obesity [30, 35, 44], epilepsy [34], heroin-dependent patients [45] and traumatic limb injury [46]. It is not only useful to compare the difference of HRQOL between cases and controls [34, 35, 38, 42] or subgroups with different severity [35] but also serves as a standard index in validation study for other disease-specific questionnaire [39, 40, 44, 46]. Therefore, WHOQOL-BREF has become an important questionnaire in QOL researches.

## Conclusions

The HRQOL for BE patients in Taiwan had poorer quality of life in the physical domain but better quality of life in the environment domain, compared to the general population. Healthcare professionals can refer to individual facets of each domain for a better clinical healthcare and management for quality of life of BE patients.

## Abbreviations

BE: Barrett’s esophagus
BMI: Body mass index
EGD: Esophagogastroduodenoscopies
ERD: Erosive reflux disease
ESEM: Endoscopically suspected esophageal metaplasia
GERD: Gastroesophageal reflux disease
HRQOL: Health-related quality of life
NERD: Non-erosive reflux disease
NHIS: National Health Interview Survey
QOL: Quality of life
TTO: Time-trade off
WHOQOL-BREF: The World Health Organization Quality of Life
